# An Update on Protein Kinases as Therapeutic Targets—Part II: Peptides as Allosteric Protein Kinase C Modulators Targeting Protein–Protein Interactions

**DOI:** 10.3390/ijms242417504

**Published:** 2023-12-15

**Authors:** Mulate Zerihun, Samuel J. S. Rubin, Shmuel Silnitsky, Nir Qvit

**Affiliations:** 1 The Azrieli Faculty of Medicine in the Galilee, Bar-Ilan University, Henrietta Szold St. 8, P.O. Box 1589, Safed 1311502, Israel; mulate.zerihun@biu.ac.il (M.Z.); shmuel.silnitsky@biu.ac.il (S.S.); 2 Department of Medicine, School of Medicine, Stanford University, 300 Pasteur Drive, Stanford, CA 94305, USA; yrubin@stanford.edu

**Keywords:** kinases, protein kinase C, allosteric, peptides, peptidomimetics, modified peptides

## Abstract

Human protein kinases are highly-sought-after drug targets, historically harnessed for treating cancer, cardiovascular disease, and an increasing number of autoimmune and inflammatory conditions. Most current treatments involve small molecule protein kinase inhibitors that interact orthosterically with the protein kinase ATP-binding pocket. As a result, these compounds are often poorly selective and highly toxic. Part I of this series reviews the role of PKC isoforms in various human diseases, featuring cancer and cardiovascular disease, as well as translational examples of PKC modulation applied to human health and disease. In the present Part II, we discuss alternative allosteric binding mechanisms for targeting PKC, as well as novel drug platforms, such as modified peptides. A major goal is to design protein kinase modulators with enhanced selectivity and improved pharmacological properties. To this end, we use molecular docking analysis to predict the mechanisms of action for inhibitor–kinase interactions that can facilitate the development of next-generation PKC modulators.

## 1. Protein Kinases

Protein kinases are a large and diverse family of more than 500 proteins encoded by ~2% of the human genome. Kinases regulate signaling networks by catalyzing the phosphorylation of specific amino acids with adenosine triphosphate (ATP) as the phosphate source, resulting in a conformational change from an inactive to an active form of the substrate or from an active to inactive conformation. Approximately one-third and up to two-thirds of the proteins in a cell may be phosphorylated at one time or another, affecting a very large set of cellular pathways by turning activities “on” or “off” [1,2]. Phosphorylation plays major roles in numerous cellular functions, including transcription, translation, metabolism, proliferation, division, cell-cycle progression, biosynthesis, movement, and survival [3]. These processes are critical to cellular homeostasis, and dysregulated kinase activity has been linked to a variety of pathological conditions, such as neurodegeneration [4], inflammation [5], autoimmunity [5], cancer [6,7,8], and cardiovascular diseases (CVDs) [9]. Imatinib (i.e., STI571, or Gleevec), which received Food and Drug Administration (FDA) approval in 2001, is considered the first protein kinase inhibitor that was clinically approved [10]. Although fasudil (an inhibitor of Rho-dependent kinases) was approved in 1995, and rapamycin (i.e., sirolimus, an inhibitor of the protein kinase TORC1) was approved in 1999, both were approved without the knowledge of the identity of their target proteins. Imatinib is a potent small-molecule kinase inhibitor (SMKI) treatment for chronic myelogenous leukemia (CML), that functions as a competitive inhibitor of the ATP-binding site. Imatinib has revolutionized drug therapy for CML, and the drug was featured on the front cover of Time Magazine (28 May 2001, Vol. 157 No. 21), termed as a “magic bullet”.

### 1.1. Protein Kinases as Major Drug Targets

Since phosphorylation plays major roles in numerous cellular functions, and it is critical to cellular homeostasis, it is not surprising that protein kinases are the second most therapeutically targeted group of proteins, after the G-protein-coupled receptors (GPCRs), and the pharmaceutical industry has dedicated approximately one-third of new drug development programs over the last decade to the development of protein kinase modulators [11,12]. Currently, there are 98 approved kinase inhibitors worldwide, 71 of which are SMKIs that have been approved by the FDA, targeting 21 kinase families constituting approximately 20% of the kinome. Interestingly, the number of SMKIs approved by the FDA has more than doubled since 2016, with 37 new approvals, making SMKIs approximately 15% of all novel drug approvals in the last 5 years (2016–2021). In addition, 16 more SMKIs have been granted approval by other regulatory agencies [13]. Currently, the majority of the FDA-approved kinase inhibitors are SMKIs targeting the kinase ATP-binding site (63 SMKIs) [14]. However, in many cases, these SMKIs demonstrate low specificity towards the target kinase, resulting in problems with toxicity and a variety of side effects, and is a major cause of clinical trial failure. Therefore, the pharmaceutical industry continues to invest in emerging trends focused on identifying alternative approaches to specifically target protein kinases more selectively.

### 1.2. Noncatalytic Domains of Protein Kinases

While the majority of studies on protein kinases focused on their catalytic activity, others demonstrated that their noncatalytic properties are also indispensable, and, in some cases, even sufficient for their effector function. Over 25 years ago, the yeast Pbs2p protein was found to serve both as a protein kinase and a scaffold protein [15]. Since then, accumulated evidence suggests that kinases possess functions beyond catalysis (noncatalytic functions), such as the scaffolding of protein complexes, allosteric regulation of other proteins through protein–protein interactions (PPIs), subcellular targeting, and deoxyribonucleic acid (DNA) binding. This diverse spectrum of activities can be used to coordinate substrate phosphorylation in a highly specific manner and support other functions that do not rely on kinase activity [16]. These noncatalytic activities involve unique interaction sites that are not as conserved as the phosphorylation site. Therefore, blocking these functions by targeting less conserved binding sites that mediate noncatalytic functions may support greater selectivity, thus reducing off-target effects [17]. These allosteric modulators also achieve high selectivity by targeting inactive kinase conformations, in which the structure does not need to be catalytically competent. Thus, each protein kinase may be targeted in a unique conformation, providing far greater opportunities for selectivity [18,19].

### 1.3. Allosteric Modulation of Kinases

The allosteric modulation approach has become an important one in drug discovery for the development of compounds that bind to sites distinct from the conserved ATP-binding site. Allosteric modulators have been used to target all major mammalian receptor superfamilies, including GPCRs, ligand-gated ion channels, and intracellular nuclear hormone receptors, providing new opportunities for basic research, as well as for therapeutic application [20,21,22]. These sites that are less conserved across the kinome and many times only available upon conformational changes provide several advantages, particularly, higher selectivity and extended drug target residence times [23]. For example, Chaikuad et al. developed the small molecule SCH772984, which is a highly specific inhibitor of extracellular signal-regulated kinase (Erk) 1 (Erk1) and Erk2. The compound targets an allosteric site, which was a previously unidentified binding pocket. The same inhibitor also binds in a completely different conformation with a lower affinity to off-target kinases [24]. An allosteric approach to target protein kinase was used by Zorba et al. for the development of monobodies (small proteins) to act either as kinase inhibitors or activators via the differential recognition of structural motifs in the allosteric pocket of the oncoprotein Aurora A (AurA) kinase. These investigators solved the crystal structure of AurA bound to activating and inhibiting monobodies, shedding light on the mechanism underlying allosteric modulation [25].

### 1.4. Use of Allosteric Sites in Drug Discovery

Many approved SMKIs demonstrate a low selectivity profile, directing the community to investigate less conserved non-ATP-binding sites. To address this issue, allosteric inhibitors targeting sites other than the orthosteric ATP-binding pocket have been developed. Allosteric kinase modulators are inhibitors that bind to an allosteric site outside the conserved ATP-binding pocket with no direct interaction with the hinge region of the ATP-binding domain, providing a significant opportunity for the generation of new classes of highly selective kinase regulators [26]. The field of allosteric kinase inhibition has evolved rapidly in the past few years with the FDA approval of the first allosteric kinase inhibitor trametinib (2018) (i.e., mekinist or meqsel). Trametinib (GSK1120212) is a unique reversible selective orally bioavailable (a mitogen-activated protein kinase (MAPK)/ERK kinase) MEK allosteric inhibitor with high affinity and nanomolar activity, which specifically binds to MEK1 and MEK2. Trametinib acts as a non-competitive ATP inhibitor that stably binds to unphosphorylated MEK and, thereby, suppresses the downstream signaling pathways involved in cell proliferation, survival, and differentiation. Trametinib demonstrated several advantages over other inhibitors, such as an improved half-life, limited toxicity, and limited interaction with other drugs. Trametinib was approved in 2013 by the FDA for the treatment of patients with V600E mutated metastatic melanoma [27].

### 1.5. Allosteric Regulation through Protein–Protein Interactions

Protein–protein interactions (PPIs) represent a significant portion of functionally relevant biological interactions and are central to most biological processes [28]. Current estimates suggest that the human repertoire of PPIs (the interactome) ranges from 130,000 to 600,000 interactions [29]. PPIs are often dysregulated in human diseases and, therefore, represent a rich source of potential therapeutic targets. Targeting PPI sites offers the potential to differentiate between many proteins and even homologous enzymes, since the sequence and/or structure of these sites are usually unique [30,31]. Targeting PPIs with small molecules is challenging, as the binding surfaces between proteins are usually large and flat and involve polar and hydrophobic interactions without a defined binding pocket. Furthermore, small molecules usually demonstrate low specificity, resulting in toxicity. Targeting PPIs with antibodies is also not straightforward, as their production can be difficult and expensive, they have low oral bioavailability, and they are usually not cell-permeable [32,33]. On the other hand, peptides and peptidomimetics (modified peptides, a term henceforth used interchangeably with “peptides” due to the overlap in defining these species) are ideal candidates to target PPIs for their unique properties, as discussed below [34,35,36,37,38].

## 2. Peptides Targeting Protein–Protein Interactions

Peptides are especially useful candidates for the inhibition of PPIs because they can mimic a protein surface to effectively compete for binding. Peptides demonstrate many advantages for targeting protein complexes compared to small molecules, such as conformational flexibility [39] and increased selectivity [40,41], thereby improving drug properties and limiting toxicity. In addition, peptides are easier and less expensive to manufacture compared to antibodies [42]. In many cases, the number of amino acids that form the PPI site and govern the binding is small (only few amino acids) [43], and it is estimated that between 15–40% of all PPIs in the cell are regulated by short linear peptides [44]. Importantly, short linear peptides derived from PPI sites can mimic the interaction site on one protein, serving as competitive inhibitors or antagonists of the respective interaction [45,46].

### 2.1. Therapeutic Application of Peptides

Peptide therapeutics have played a notable role in medical practice since the isolation and commercialization of insulin [47], which was the first peptide to be administered therapeutically. Currently, peptides are used for a wide range of indications, including metabolic disease, infectious disease, neurological disease, autoimmune diseases, oncology, CVDs, and a variety of other disorders [48,49]. The number of peptides entering clinical studies continues to grow, from 1.2 per year (1970s) to over 16.8 per year (2000s) [50,51]. Peptides demonstrate superior success rates in transitioning from phase 1 to phase 2 trials (83%) compared to small molecules and biological drugs (63% and 77%, respectively) and in transitioning from phase 3 to regulatory review (68% compared to 61% for small molecules and 63% for biological drugs) [52]. Aside from crossing the cell membrane independently (e.g., cyclosporine), peptides and peptidomimetics can be conjugated to cell-penetrating vehicles to modulate intracellular targets [53]. The coupling of cargo to cell-permeable peptides has been used extensively to deliver molecules, peptides, and proteins in cells [54], animal models [55], and humans [56] (for a review, see [57]). It is not surprising that the number of available therapeutic peptides is increasing, and, as of 2020, there were >100 approved peptides and peptidomimetics with therapeutic or diagnostic applications on the market. About 155 peptides are in clinical trials, and over 500 are in preclinical development. In addition, four peptides reached global sales of over $1 billion as early as 2010, including glatiramer acetate ($4.0 billion), leuprolide acetate ($3.0 billion), octreotide acetate ($1.3 billion), and goserelin acetate ($1.1 billion) [58,59,60,61].

### 2.2. Approaches to Developed Peptides as Protein Kinase Regulators Targeting Allosteric Sites

There are several approaches to identify peptides that target PPIs, including rational design methods, as well as screening large peptide libraries in which peptides can be designed using systematic or random methodologies. The major approaches that use large screen methodologies include the orderly search of large domains involved in PPIs, random search of large domains involved in PPIs, and search of key amino acid residues involved in PPIs. While all the above approaches were used successfully to identify peptides that regulate PPIs, they are labor-intensive and costly, as they require the use of large libraries, limiting their practical use. Herein, we will discuss several rational approaches to develop peptides that target kinase PPIs by allosteric modulators: (1) Peptides derived from unique substrate sites, demonstrating high selectivity toward the inhibition of their corresponding kinase. For example, peptides mimic the pseudo-substrate sequence, thereby directly disrupting the phosphorylation reaction by competing with substrates, resulting in the inhibition of kinase activity. This approach has proven successful for the inhibition of actions of specific isoforms of protein kinase C (PKC) [62,63]. (2) Peptides derived from similarity sequences in binding proteins. For example, it was demonstrated that short homologous sequences between unrelated proteins may represent the binding sites of some signaling enzymes, and peptides that derived from these sequences were demonstrated to regulate the enzyme PPI [40,64]. (3) Peptides derived from evolutionarily conserved sequences. In many cases, it was demonstrated that evolutionarily conserved components play a critical and vital role in signal transduction, as well as cell homeostasis. Therefore, peptides derived from these sequences were demonstrated to regulate enzyme activity, as well as cell function [65,66,67,68]. (4) Peptides derived from unique sequences of the protein kinase. An interesting and opposing approach identified non-conserved domains, or domains that differ from each other, in kinase isozymes to develop peptides that are specific for one isozyme [69].

## 3. Peptides Derived from Unique Substrate Sites

A peptide derived from a unique site of recognition of the substrate may demonstrate a high selectivity toward regulating the activity of its corresponding kinase. As an example, peptides mimicking the pseudo-substrate sequence confer increased selectivity. The pseudo-substrate is a key molecular switch in PKC isozyme regulation. This sequence contains a stretch of basic amino acids that is similar to the consensus substrate sequence, but it has an Ala at the position of the phosphor-acceptor. These peptides directly disrupt phosphorylation by competing with substrates and inhibiting the activity of the kinase enzyme. Through the specific disruption of each PKC isoform’s binding proteins with its intracellular-binding proteins, this approach has proven successful in inhibiting specific PKC isoforms [70,71].

### 3.1. Peptides Derived from the Pseudo-Substrate Site

PKC enzymes contain an autoinhibitory domain, termed the pseudo-substrate domain, which is embedded in the N-terminal regulatory region and binds a catalytic domain sequence to inhibit kinase activity. The autoinhibitory domain is lodged in the kinase active site, preventing it from phosphorylating substrates. The pseudo-substrate domain comprises a stretch of basic amino acids resembling the consensus substrate sequence but with an Ala at the position of the phosphor-acceptor site. The peptide PKC 19-36 is an α/β pseudo-substrate derived from the protein sequence RFARKG**A**LRQKNVHEVKN (Table 1). House et al. synthesized the same peptide with Ser instead of Ala (i.e., RFARKGA(→S)LRQKNVHEVKN), which acted as a PKC substrate [72]. Peptides derived from the pseudo-substrate region show an autoinhibitory effect on PKC activity and are attractive PKC inhibitors. These peptides were initially used to demonstrate the autoinhibitory role of this domain, which made them attractive candidates for use as PKC inhibitors within a cellular context. In many cases, these peptides are considered specific PKC inhibitors and are widely used in various forms [73]. Numerous analogs of this peptide have been developed and evaluated in preclinical and clinical assays, including peptides derived from PKCα_15-28_ [74], PKCα_20-28_ [75], and PKC_19-36_ [76,77], to mention a few.

**Figure 1:**
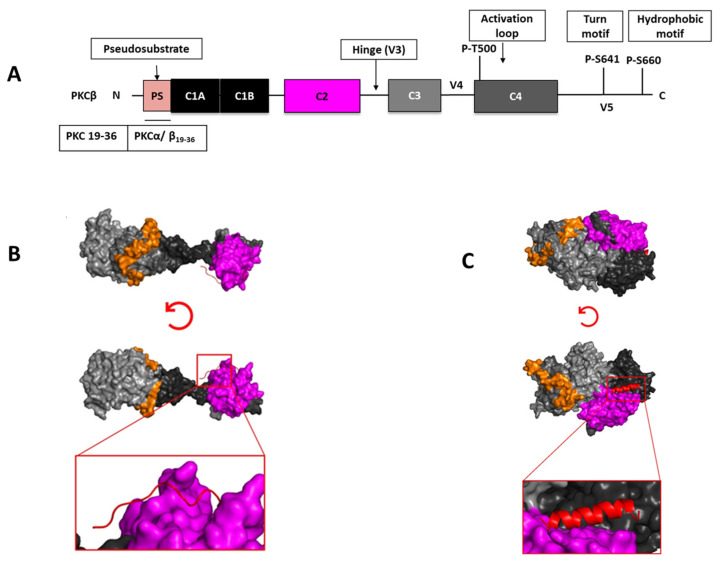
Structural domains of PKCβ and peptides derived from the pseudo-substrate site. The C1 region of PKC contains the pseudo-substrate (PS) sequence (amino acid residues 19–36) that is thought to maintain the enzyme in a conformationally restricted resting state. Pseudo-substrate peptides that are derived from the autoinhibitory domains that lie outside the kinase catalytic domain were designed to disrupt interactions between protein kinases and their own A-loop [84]. (**A**) Schematic representation of full-length PKCβ structural elements. PKCβ contains a C1 domain (black), C2 domain (magenta), C-lobe (gray), N-lobe of the kinase domain (orange), and C3 and C4 domains (grey). (**B**,**C**) Molecular docking results for the interaction of PKC 19-36 peptide (RFARKGALRQKNVHEVKN) and PKCβ to the (**B**) crystal structure (PDB: 3PFQ [78]) or (**C**) AlphaFold predicted model (P05771) are shown. PKCβ is shown by cartoon representation colored in (C1 domain—black, C2 domain—magenta, and V5 domain—orange), and the peptide is shown in red cartoon structure. Magnified view of peptide binding (red cartoon) to PKCβ C1 and C2 domains. PyMol (Schrodinger LLC, New York, NY, USA) was used to generate the figure [85]. The docking was carried out with HPEPDOCK 2.0 [86].

We performed docking analyses between the PKC 19-36 peptide RFARKGALRQKNVHEVKN and PKCβ (using the available crystal structure (PDB: 3PFQ [78]) and the AlphaFold predicted model (P05771)). PKC 19-36 binds to the C1 domain at the interface between the C1 and C2 domains. The importance of these domains in PPIs has been demonstrated in many studies [79,80]. Furthermore, Stahelin et al. demonstrated the importance of “tethering” between the C1 and C2 domains [81]. In addition, the C1A of PKCβ was critical for protein interactions with other binding partners (e.g., pericentrin, a scaffold protein of the centrosome) [82]. Thus, these docking results are consistent with current experimental data (Figure 1).

**Table 1:** Peptides derived from the pseudo-substrate site.

Peptide	Parent Protein_AA-AA_	Protein Accession #	Peptide Sequence	Peptide Role
PKC 19-36	PKCα/β_19-36_	P17252/P05771	RFARKG**A**LRQKNVHEVKN	**Antagonist**: the peptide inhibited PKC function [83]

Amino acids are represented by their one-letter code.

### 3.2. Peptides Derived from Substrate Phosphorylation Sites

Additional peptides have been derived from substrate amino acid sequences including nuclear factor related 2 (Nrf2), myelin basic protein (MBP), and hepatitis C virus (HCV) nonstructural protein 3 (NS3). Nrf2 is a basic leucine zipper transcription factor containing seven potential PKC phosphorylation sites. Huang et al. developed peptides that mimic these sites and demonstrated that Nrf2 phosphorylation by PKC was reduced by more than 90% in the presence of a particular peptide; using a site-directed mutagenesis approach, they also found that Ser40 is the PKC substrate and the only PKC binding site on Nrf2 [87]. MBP is also a PKC substrate, and the peptide MBP 104-118 was shown to specifically inhibit PKC. A modification of one amino acid (either Arg to Ala at position 107 or Arg to Ala at position 113) inhibited the protein phosphorylation of intact MBP [88,89]. The HCV NS3 protein inhibits nuclear transport and the enzymatic activity of the protein kinase A (PKA) catalytic subunit. Borowski et al. developed a peptide termed HCV (1487-1500), which reproduces the PKA-binding domain of NS3. The peptide directly interacts with the kinase and serves as its substrate. The authors speculated that the arginine-rich sequence interacts with the catalytic domain of PKC, as observed in the case of PKA in vivo [90], and suggested that the peptide could be used to manipulate PKC function. They ultimately found that NS3 affects PKC function by a complex mechanism similar to that which inhibits PKA (Table 2) [91].

ElK-1 is a transcription factor that plays an important role in regulating ERK target genes in the suprachiasmatic nucleus (SCN) in response to light, especially core oscillator genes. Myeloid zinc finger 1 (MZF-1) is a multifaceted transcription factor that may function as either an oncogene or a tumor suppressor, and the molecular bases determining its different traits remain elusive. Hsieh et al. demonstrated that both ElK-1 and MZF-1 transcription factors form a heterodimer and may be the critical regulators of PKCα [92]. Based on these findings, Yue et al. developed a peptide termed MZF-1, which was derived from the PKC-binding sites for Elk-1 and MZF-1. Treatment with the peptide resulted in the inhibition of AXL expression, a tyrosine kinase that is overexpressed in triple-negative breast cancer (TNBC) and plays a role in PKCα downregulation and, ultimately, tumor progression and metastases (Table 2) [93].

Tyrosinase is a copper-containing enzyme widely distributed in different organisms and catalyzes key reactions in melanin biosynthesis. PKCβ activates tyrosinase by phosphorylating Ser505 and Ser509 [94], and the loss of PKCβ prevents melanogenesis in cultured pigment cells, suggesting that PKCβ inhibition might lead to skin and hair lightening in vivo. Park et al. developed a tyrosinase mimetic peptide (TMP) to inhibit the PKCβ phosphorylation of tyrosinase, resulting in reduced tyrosinase activity. The peptide is derived from the PKCβ phosphorylation site on tyrosinase, and it inhibits the phosphorylation of tyrosinase by PKCβ. However, the authors indicate that the amino acid sequence of TMP deviates considerably from the substrate sequence of XRXXSXRX (where X is any amino acid), which is common in the classic PKC isozymes. Therefore, it remains to be determined whether TMP is a pseudo-substrate only for PKCβ [95].

Based on the phosphorylation site domain in myristoylated alanine-rich C-kinase substrate (MARCKS) containing a basic region of 25 amino acids with four conserved serines, Graff et al. developed a peptide termed **p**hosphorylation **s**ite **d**omain (PSD) peptide, which was phosphorylated by PKC with high affinity. Upon replacement of all serines by alanines, the peptide inhibited the PKC phosphorylation of histone and peptide substrates. The tetra-alanyl peptide acted as a potent inhibitor of PKC, although not specific for PKC [96,97].

Jayaram et al. identified an upstream open reading frame (uORF)-encoded peptide that regulates PKCη, which also includes the PKC pseudo-substrate motif. This uPEP2 peptide directly binds to and selectively inhibits the catalytic activity of novel PKCs. The treatment of breast cancer cells with the peptide diminished cell survival and migration and synergized with chemotherapy by interfering with the response to DNA damage. In a xenograft mouse model of breast cancer, the peptide also suppressed tumor progression, invasion, and metastasis [98].

PKC catalyzes the phosphorylation of the epidermal growth factor (EGF) receptor (EGFR) at Thr654. An N-myristoyl-octapeptide derived from this sequence exhibited potent inhibitory activity against PKC; however, neither the myristic acid nor the non-myristylated peptide were active [99,100]. A pentapeptide based on the EGFR phosphorylation site KRTLR acted as a PKC substrate [101], and its corresponding N-myristoyl-KRTLR species behaved as a PKC antagonist [102]. Thus, the aforementioned studies illustrate that novel compounds derived from substrate phosphorylation sites can modulate target protein phosphorylation.

**Table 2:** Peptides derived from substrate phosphorylation sites.

Peptide	Parent Protein_AA-AA_	Protein Accession #	Peptide Sequence	Peptide Role
Nrf2	Nrf2_35-44_	Q16236	VFDFSQRQ	**Antagonist**: the peptide inhibited PKC function [87].
MBP 104-118	MBP_238-252_	P02686	GKGRGLSLSRPSWGA	**Antagonist**: the peptide inhibited PKC function [88].
HCV (1487-1500)	NS3_1487-1500_	Q68866	RRGRTGRGRRGIYR	**Antagonist**: the peptide inhibited PKC function [91].
MZF-1	MZF-1_309-321_	P28698	SDLRSEQDPTDED	**Antagonist**: the peptide inhibited PKC expression [93].
TMP	Tyrosinase_501-511_	P14679	EDYHSLYQSHL	**Antagonist**: the peptide inhibited PKC function [95].
PSD peptide	MARCKS_86-110_	P49006	KKKKKRF**A**FKK**A**FKL**A**GF**A**FKKNKK	**Antagonist**: the peptide inhibited PKC function [96].
Octapeptide	EGFR_675-682_	P00533	RKR**T**LRRL	**Antagonist**: the peptide inhibited PKC function [103].
uPEP2	uORF2_1-26_	C0HM02	MASRGALRRCLSPGLPRLLHLSRGLA	**Antagonist**: the peptide inhibited PKC function [98].

Amino acids are represented by their one-letter code.

### 3.3. Peptides Derived from Substrate Protein–Protein Interaction Sites

Distal docking sites are a vital mechanism of specificity between docking motifs on the substrate and interaction domains on the kinase [104]. Docking sites are used to tether the substrate distal to the active site and increase the local concentration of phosphor-acceptor sites, leading to enhanced kinase affinity for specific substrates. Docking interactions have been identified for various kinases, including ERK [105], MEK [106,107], glycogen synthase kinase-3 (GSK3) [108], phosphoinositide-dependent kinase-1 (PDK-1) [109], cyclin-dependent kinase-2 (CDK2) [110,111], and many others, emphasizing the generality of this mechanism [112,113].

**Figure 2:**
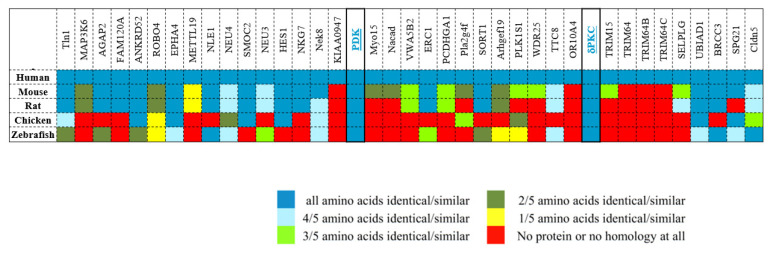
ψPDK peptide sequence specificity. There are 39 human proteins with ψPDK sequences. The heat-map shows amino acid sequence conservation in orthologues of these proteins. Sequence conservation is observed only in PDK and δPKC. Adapted from [55].

A series of peptides targeting PKCδ and substrates was developed and evaluated in various animal models. For example, Qvit et al. developed an inhibitor of phosphorylation for only one PKC substrate. In this study, a distal docking site on PKCδ was identified that interacts with the substrate pyruvate dehydrogenase kinase (PDK, a different protein than phosphoinositide-dependent kinase-1 (PDK-1)). The peptide ψPDK was derived from the C2 domain of PKCδ, which is highly similar to PDK and conserved in all the four isoforms of PDK. While other human proteins contain the ψPDK peptide sequence, it is not conserved in other species, suggesting that it is only functionally significant in δPKC and PDK (Figure 2). The peptide inhibited PDK phosphorylation without affecting the phosphorylation of the other PKCδ substrates, even at 1 μM, with an in vitro K_D_ of ~50 nM. The peptide reduced cardiac injury with an IC_50_ of ~5 nM, demonstrating that PDK phosphorylation is critical for PKCδ-mediated cardiac injury [55].

A similar approach was used to design other inhibitors based on PKCδ substrates, including glyceraldehyde-3-phosphate dehydrogenase (GAPDH), an important glycolytic enzyme that has a noncatalytic (noncanonical) role in mitochondrial elimination under oxidative stress associated with increased cellular injury. The peptide ψGAPDH is derived from a short sequence in the V3 domain of PKCδ that is similar to GAPDH, and it reduced GAPDH glycolytic activity in vitro and ex vivo. ψGAPDH also inhibited the elimination of damaged mitochondria [114].

The sequence alignment of PKCδ with three additional substrates identified the following peptides: (i) ψMARCKS derived from the PKCδ and MARCKS PPI site; (ii) ψDrp1 derived from the PKCδ and dynamin related protein 1 (Drp1) PPI site; and (iii) ψIRS1 derived from the PKCδ and insulin receptor substrate 1 (IRS1) PPI site. The peptides bound to PKCδ with high affinity (low nanomolar range) but not to other PKC isozymes such as PKCε. Furthermore, ψDrp1 and ψIRS1 demonstrated high specificity for inhibiting the phosphorylation of their corresponding substrate in vitro, in cell culture, and in an animal model demonstrating reduced cardiac injury [54]. A selective inhibitor was also developed for cardiac troponin I (cTnI), a sarcomere protein key for cardiomyocyte contraction that is phosphorylated by PKCδ. The peptide ψTnI derived from PKCδ and cTnI inhibited the cTnI interaction with and phosphorylation by PKCδ, preventing tissue injury in an ex vivo model of myocardial infarction and attenuating ischemia-reperfusion injury-induced mitochondrial dysfunction [115]. pAnxV derived from annexinV and PKCδ was developed with an amino acid charge difference characteristic of a PKC-RACK (receptor for activated C kinase) relationship (Glu to Arg). This RACK-like sequence in annexin V is not found in other members of the annexin family. pAnxV also inhibited PKCδ function and translocation from the cellularly soluble to the cellularly precipitate fraction in a model of myocardial infarction (Table 3) [116].

**Figure 3:**
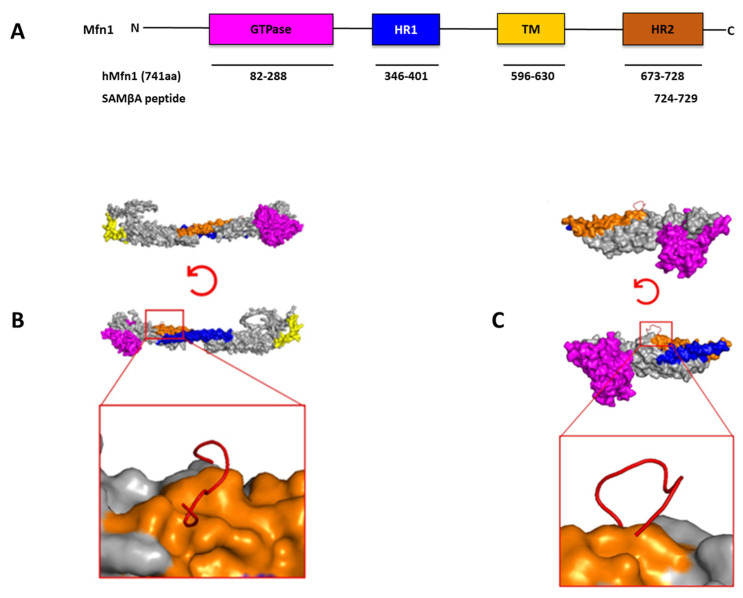
Structural domains of Mfn1 and peptides derived from substrate protein–protein interaction sites. A sequence corresponding to the homologous domain between PKCβII (P05771-2; residues 625–629) and Mfn1 (26251799; residues 724–729) was identified, and the SAMβA peptide corresponding to this sequence was developed. (**A**) The structural and functional domains of full-length mitofusin-1 (Mfn1) (741 AAs). The GTPase domain is shown in magenta, heptad repeat (HR1) coiled-coil regions 1 in blue, the transmembrane (TM) domain is shown in yellow, and heptad repeat (HR2) coiled-coil regions 2 in orange. (**B**) Molecular docking results for the interaction of SAMβA (RNAENFDRF) and Mfn1 to the available crystal structure (PDB: 5GO4 [118]) or (**C**) AlphaFold predicted model (Q8IWA4). To help stabilize the α-helix structure, amino acids were added at the C- and N-terminus of the peptide. Mfn1 is shown by cartoon representation colored in (GTPase—magenta, HR1—blue, HR2—orange, TM—yellow) and the peptide is shown in red cartoon structure. Based on the docking analysis, the peptide is docked to the HR2, which is source domain for its rational design. PyMol (Schrodinger LLC, New York, NY, USA) was used to generate the figure [85]. The docking of SAMBA to both Mfn1 structures was carried out with HPEPDOCK 2.0 [86].

The same approach was also used to target mitofusin 1 (Mfn1), a GTPase homologous protein that is localized to the outer mitochondrial membrane where it mediates mitochondria fusion as a downstream PKCβII substrate involved in heart failure pathophysiology. A selective inhibitor termed SAMβA (**s**electively **a**ntagonizes **M**fn1-PKC**β**II **a**ssociation) derived from the PKCβII V5 domain and Mfn1 was developed and shown to protect cultured neonatal and adult cardiac myocytes, but not Mfn1 knockout cells, from stress-induced cell death. The treatment with the inhibitor restored mitochondrial morphology and function and improved cardiac contractility in an animal model of heart failure (Table 3) [117]. We performed docking analyses of the SAMβA peptide RNAENFDRF to Mfn1 using the available crystal structure (PDB: 5GO4 [118]) or AlphaFold predicted model (Q8IWA4). The peptide docked to the HR2, which is the source domain for its rational design (Figure 3).

Heat shock protein 90 (HSP90) is a molecular chaperone and a cytoprotective protein that participates in the mitochondrial import of several proteins including PKCε. A peptide was derived from PKCε and HSP90 where there are two charge differences (Lys140 and Asn142 on PKCε versus Glu553 and Glu555 on HSP90), which may be important for their interaction [116,119]. Treatment with the ψεHSP90 peptide enhanced PKCε and HSP90 interactions, enhanced PKCε mitochondrial translocation, increased the phosphorylation and activity of a mitochondrial PKCε substrate, and reduced cardiac injury in animal models of myocardial infarction (Table 3) [120].

**Table 3:** Peptides derived from substrate protein–protein interaction sites.

Peptide	Parent Protein_AA-AA_	Protein Accession #	Peptide Sequence	Peptide Role
ψPDK	PKCδ_36-40_ homolog to PDK_391-395_	Q05655 Q15119	ALSTE ::::. ALSTD	**Inhibitor**: the peptide inhibited PKCδ binding to PDK [55]. **Antagonist**: the peptide inhibited PKCδ function [55].
ψGAPDH	PKCδ_311-316_ homolog to GAPDH_169-174_	Q05655 P04406	GIYQGF ::..: GIVEGL	**Inhibitor**: the peptide inhibited PKCδ binding to GAPDH [114]. **Antagonist**: the peptide inhibited PKCδ function [114].
ψMARCKS	PKCδ_75-80_ homolog to MARCKS_256-261_	Q05655 P29966	RAAEEP .::::: KAAEEP	**Inhibitor**: the peptide inhibited PKCδ binding to MARCKS [54]. **Antagonist**: the peptide inhibited PKCδ function [54].
ψDrp1	PKCδ_630-635_ homolog to Drp1_101-106_	Q05655 O00429	YSNFDQ :..::. YTDFDE	**Inhibitor**: the peptide inhibited PKCδ binding to Drp1 [54]. **Antagonist**: the peptide inhibited PKCδ function [54].
ψIRS1	PKCδ_620-626_ homolog to IRS1_264-270_	Q05655 P35568	FRPKVKS :::. :: FRPRSKS	**Inhibitor**: the peptide inhibited PKCδ binding to IRS1 [54]. **Antagonist**: the peptide inhibited PKCδ function [54].
ψTnI	PKCδ_54-61_ homolog to cTnI_190-197_	Q05655 P19429	EWKSTFDA .:. .:: DWRKNIDA	**Inhibitor**: the peptide inhibited PKCδ binding to troponin [115]. **Antagonist**: the peptide inhibited PKCδ function [115].
pAnxV	AnnexinV_157-164_ homolog to PKCδ_74-81_	P08758 Q05655	QANRDP :::::: QANRDP	**Inhibitor**: the peptide inhibited PKCδ binding to AnnexinV [116]. **Antagonist**: the peptide inhibited PKCδ translocation and function [116].
SAMβA	PKCβII_625-629_ homolog to Mfn1_724-729_	P05771-2 26251799	N-AENF : ::: NELENF	**Inhibitor**: the peptide inhibited PKCβII binding to Mfn1 [117]. **Antagonist**: the peptide inhibited PKCβII translocation and function [117].
ψεHSP90	PKCε_139-145_ homolog to HSP90α_552-558_	Q02156 NP_005339	PKDNEER :.:.::. PEDEEEK	**Activator**: the peptide increased PKCε binding to HSP90 [120]. **Agonist**: the peptide increased PKCε translocation and function [120].

Amino acids are represented by their one-letter code; “:” indicates identical amino acids; and “.” indicates conserved amino acid substitutions.

## 4. Peptides Derived from Similar Sequences in Binding Proteins

In some cases, signaling enzymes interact with multiple proteins that are unrelated to one another. In many cases, these unrelated proteins share a short homology sequence that could represent the enzyme-binding site. Consequently, peptides corresponding to that sequence may interfere with enzyme binding and/or activity [121]. For example, A-kinase anchor proteins (AKAPs) act as scaffolding proteins that tether PKA. These proteins act as spatial and temporal PKA regulators by localizing PKA along with multiple proteins into discrete signaling complexes. Carr et al. developed a peptide, Ht31, derived from canonical docking interactions between AKAPs and PKA. Ht31 binds the PKA regulatory subunit type II (RII) with high affinity. This prevents PKA from interacting with AKAPs [122].

### 4.1. Peptides Derived from Sequences Shared by Non-Related Proteins That Interact with a Common Protein

In many cases, PPIs are mediated through specifically recognized short motifs on the protein surface, which exhibit characteristics needed to ensure binding specificity [123,124]. Frequently, enzymes interact with multiple non-related proteins (e.g., a kinase with several substrates), and these short sequences homologous between non-related proteins can represent binding sites for their interaction partners [79]. Thus, peptides that are derived from these sequences may modulate the PPI, resulting in effects on enzyme activity and/or function. A classic example was identified over 20 years ago by Dintilhac et al. using a large library of hepta-peptides displayed on phages. This work identified a linear sequence of homology to other proteins in the high-mobility group protein 1 (HMGB1) as a potential recognition motif of these proteins to HMGB1. Based on this sequence, they predicted new proteins that interact with HMGB1, and, using a pull-down assay, they confirmed the binding [123].

**Figure 4:**
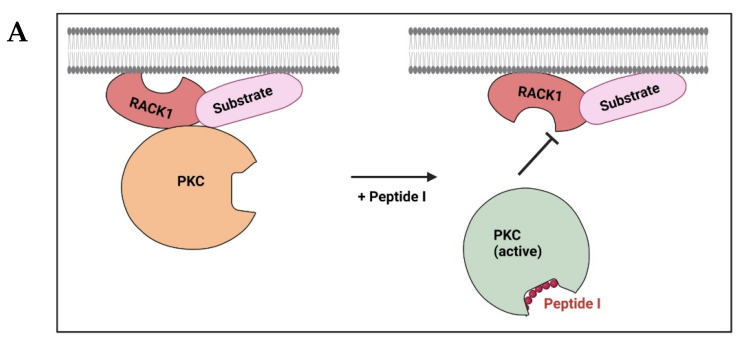

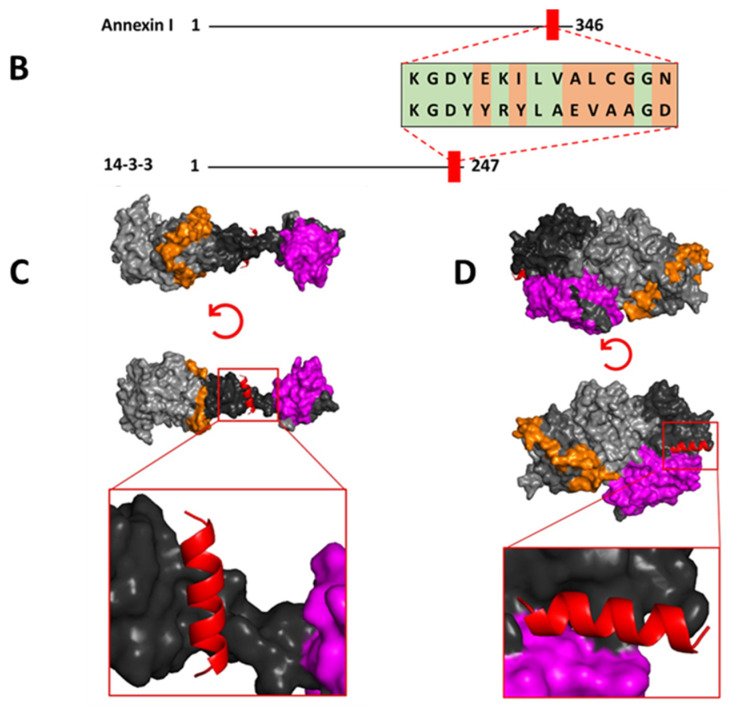
Rationally designed peptides modulating PKC interactions with RACK1, and PKC substrates (e.g., annexin I and 14-3-3). Peptides were derived from sequences shared by non-related proteins that interact with a common protein. (**A**) Peptide I derived from homology sequences between annexin 1 and 14-3-3 inhibits PKC-RACK1 interactions as well as PKC translocation. This figure was created using BioRender.com (accessed on 1 October 2023). (**B**) A sequence corresponding to the homologous domain between two PKC-binding proteins, Annexin I (P04083; residues 332–346) and 14-3-3 (P63104; residues 122–136), was identified, and peptide I corresponding to this sequence was developed. (**C**,**D**) Molecular docking results for the interaction of peptide I, KGDYEKILVALCGGN, and PKCβ to the (**C**) crystal structure (PDB: 3PFQ [78]) and (**D**) AlphaFold predicted model (P05771) are shown. PKCβ is shown by cartoon representation colored in (C1 domain—black, C2 domain—magenta, and V5 domain—orange) and the peptide is shown in red cartoon structure. PyMol (Schrodinger LLC, New York, NY, USA) was used to generate the figure [85]. Scheme is not drawn to scale.

Ron et al. proposed that a short sequence of homology in 14-3-3 (protein kinase C inhibitor protein 1, or KCIP-1) and annexin I identified previously [125] might be the PKC-binding site of these non-related proteins that both interact with PKC. To further establish this hypothesis, they developed peptide, termed peptide I, which is based on that short sequence. Peptide I inhibits PKCβ translocation and function in vivo as it binds the activated PKC and inhibits its interaction with the anchored receptor for activated C kinase 1 (RACK1) [126,127]. Next, they aligned the sequences of 14-3-3 and annexin I with RACK1 and identified two short sequences of homology between the interacting partners. Based on these motifs, they developed two additional peptides, peptide RACK1-rIII derived from the homology between 14-3-3 and RACK1, and peptide RACK1-rVI derived from the homology between annexin I and RACK1. Both peptides inhibited PKC binding to RACK1. While peptide I and peptide RACK1-rVI inhibited PKC binding to RACK1, they exhibited opposite activities in an oocyte model. Peptide I inhibited, whereas peptide RACK1-rVI stimulated insulin-induced *Xenopus* oocyte maturation, and a similar affect was observed for PKC translocation. Although both peptides had sequence homology, one mimicked the hormone-induced PKC-mediated function while the other inhibited this hormone-induced function. The authors suggested that PKC undergoes transient spontaneous conformational changes required for activation and that the peptide RACK1-rVI stabilizes this conformation. Specifically, they speculated that the peptide binds the inactive form of PKC and induces conformational changes required for the activation and translocation of the enzyme in vivo. Finally, the authors found that peptide I and peptide RACK1-rVI bind the same site on PKC and suggested that the opposite charge difference between the peptides (peptide RACK1-rVI contains Asp and peptide I contains Lys) may induce a conformational change in PKC with opposing consequences on the biological activity of the enzyme (Table 4; Figure 4) [127,128].

We performed docking analyses of peptide I, KGDYEKILVALCGGN, to PKCβ (to the available crystal structure (PDB: 3PFQ [78]) and to the AlphaFold predicted model (P05771)). The peptide binds to the C1 domain near the interface of the C1 and C2 domains. Many studies demonstrated the importance of these domains in PPIs [79,80]. The C1b domain of PKCγ bound 14-3-3τ and was shown to be critical in the regulation of solute transport across Gap junctions [129]. Thus, these peptide docking results align with the current experimental knowledge.

**Table 4:** Peptides derived from sequences shared by non-related proteins that interact with a common protein.

Peptide	Parent Protein_AA-AA_	Protein Accession #	Peptide Sequence	Peptide Role
Peptide I	Annexin A1_332-346_ homolog to 14-3-3_122-136_	P04083 P63104	KGDYEKILVALCGGN ::::.:.:. . : KGDYYRYLAEVAAGD	**Inhibitor**: the peptide inhibited PKCβ binding to RACK [130]. **Antagonist**: the peptide inhibited PKCβ translocation and function in vivo [127].
RACK1-rIII	RACK1_107-113_ homolog to 14-3-3_42-48_	P63244 P04083	DVLSVAF .::::. NLLSVAY	**Inhibitor**: the peptide inhibited PKCβ binding to RACK [128].
RACK1-rVI	RACK1_234-241_ homolog to Annexin A1_337-344_	P63244 P04083	**D** IINALCF .:. ::: **K** ILVALCG	**Inhibitor**: the peptide inhibited PKCβ binding to RACK [127,128]. **Agonist**: the peptide induced PKCβ autophosphorylation and substrate phosphorylation [127,128].

Amino acids are represented by their one-letter code; “:” indicates identical amino acids; and “.” indicates conserved amino acid substitutions.

### 4.2. Peptides Derived from Sequences Involved in Intramolecular Interactions

Pseudo-RACK (ψRACK) is a peptide that mimics the RACK-binding site, which binds to PKC with a lower affinity than intact RACK. ψRACK sequence motifs similar to intact RACK PKC-binding sites were identified in PKCβ, PKCδ, and PKCε. A single charge change lowers the affinity of the intramolecular interaction, presumably allowing the displacement of the RACK sequence and favoring PKC binding to its RACK protein upon PKC activation. For the PKCβ sequence _241_SV**E**IWD^246^ and the RACK1 sequence _255_SI**K**IWD^260^, the single mutation of SVE(→K)IWD generates a sequence more closely mimicking the binding sequence at the anchor protein [131]. For the PKCδ sequence _71_IVLMRRA**E**DPMSE^83^ and the annexin V sequence _154_VVLLQAN**R**DPDAG^166^, a single mutation of IVLMRRAE(→R)DPMSE generates a sequence more closely mimicking the binding sequence at the anchor protein [119]. For the PKCε sequence _85_H**D**APIGYD^92^ and the β′-COP sequence _285_N**N**VALGYD^292^, a single mutation of HD(→N)APIGYD generates a sequence more closely mimicking the binding sequence at the anchor protein (Table 5) [119].

**Figure 5:**
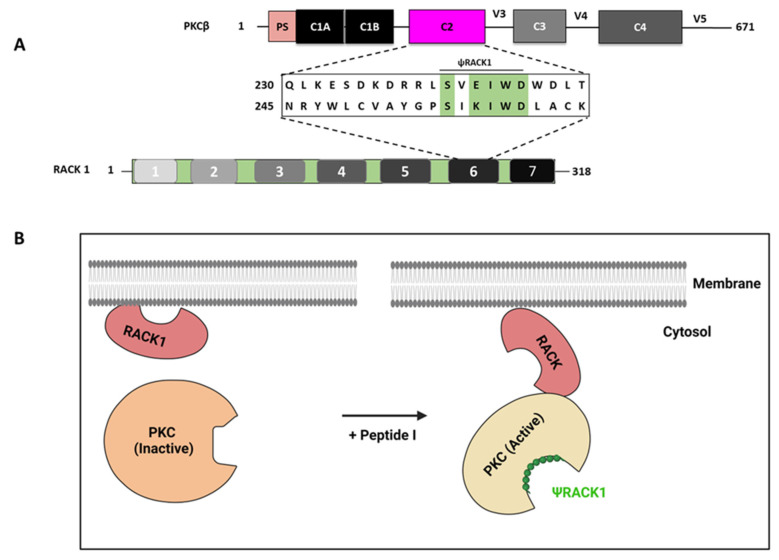
Peptides derived from sequences involved in intramolecular interactions. (**A**) A sequence corresponding to the homologous domain between PKCβ (P05771; residues 241–246) and RACK1 (P63244; residues 255–260) was identified, and the ψRACK1 peptide corresponding to this sequence was developed. (**B**) Shown are receptors for activated C kinase (RACK)—specific anchoring proteins for a PKC isozyme and corresponding to PKC isozyme. ψRACK1 peptide binds and activates PKC and, thereby, acts as an agonist. The schemes are not drawn to scale. This figure was created using BioRender.com (accessed on 1 October 2023).

The RACK receptor specific for activated PKCβ, RACK1, has short sequences of homology to PKCβ. Ron et al. hypothesized that the homologous RACK1 sequences found in PKC may serve as autoregulatory regions between PKC, the ligand, and RACK1, its receptor. The authors developed two peptides: one derived from the C2 domain of PKCβ corresponding to ψRACK1, and one derived from the sixth WD40 repeat of RACK1, which they termed the RACK1-derived peptide. They hypothesized that peptides corresponding to the ψRACK site act as allosteric agonists by interfering with the autoinhibitory intramolecular interaction between the ψRACK site and the RACK-binding site within PKC, thus stabilizing a state of PKC in which the RACK-binding site is available for PPIs, enabling the association of the enzyme with its anchoring RACK. ψRACK1, but not the RACK1-derived peptide, modulated PKC function both in vitro and in vivo. The ψRACK1 peptide binds and activates PKC in the absence of PKC activators and, thereby, acts as an agonist of PKC function in vivo (Table 5; Figure 5) [131].

Dorn et al. reported a PKCε-selective agonist octapeptide called ψεRACK, which was derived from the C2 domain of the PKCε motif homologous to its anchoring protein εRACK (i.e., β′-COP). Treatment with ψεRACK increased PKCε translocation and protected cardiomyocytes in a model of ischemia without any deleterious effects [132]. A follow-up study demonstrated that changing the charge of the ψεRACK peptide through a substitution of Asp with Asn in the sequence HD(→N)APIGYD produced opposing activity, and the substitution of Asp with Ala in the sequence HD(→A)APIGYD resulted in an inactive peptide [133]. A similar rationale guided the design of ψδRACK, a PKCδ-selective agonist (Table 5) [119].

## 5. Evolutionarily Conserved Peptides

Many signaling enzymes have homologous domains that perform similar functions, yet have unique functions. Conserved sequences within such domains may be essential for the function. These conserved sequences are expected to serve the same function in proteins from evolutionarily distant organisms. Consequently, peptides corresponding to that sequence may interfere with enzyme binding and/or activity. For example, the C2 domain is a calcium-dependent membrane-targeting module found in many cellular proteins involved in signal transduction or membrane trafficking. It is the second most abundant lipid-binding domain found, and it mediates protein–protein interactions. Peptides derived from a homologous sequence of the C2 domain from evolutionarily distant organisms act as isozyme-selective regulators, demonstrating high binding and bioactivity [132,134].

### 5.1. Peptides Derived from Evolutionarily Conserved Sequences

Amino acids directly involved in protein function tend to be more conserved over evolutionary time than other residues, and the conservation level of specific residues generally informs how important a given residue is to protein structure and/or function [135,136,137]. Using this rationale, Johnson et al. developed the selective PKCε antagonist εV1-2, which was derived from the PKCε C2 domain that binds to RACK2 (previously identified as β′-COP). εV1-2 is selective for PKCε and inhibits PKCε translocation and function in cardiac myocytes, and it was designed based on a sequence conserved between *Homo sapiens* and *Aplysia californica* (slug). The peptide abolished hypoxic preconditioning and phorbol-ester-mediated cardiac protection, suggesting that the activation of PKCε is critical for cardiac myocyte protection (Table 6). Another peptide termed ψεRACK was designed using a similar approach discussed above [134,138]. An additional study using biophysical surface plasmon resonance (SPR) and nuclear magnetic resonance (NMR) indeed confirmed that εV1-2 bound to β′-COP and inhibited PKCε binding. In the same study, they also found that ψεRACK did not bind to PKCε, suggesting that their mechanisms of action may be different [139].

### 5.2. Peptides Derived from Conserved Sequences in Homologous Domains of Otherwise Non-Related Proteins

**Figure 6:**
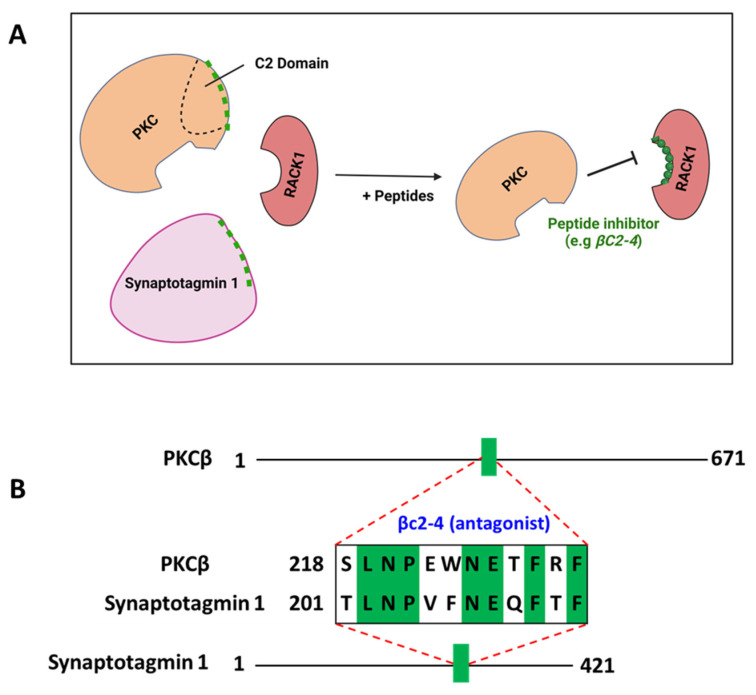
Peptides derived from conserved sequences in homologous domains of otherwise non-related proteins. (**A**) Peptides βC2-4 derived from homology sequences between PKCβ and synaoptotagmin-1 inhibit PKC-RACK1 interactions. (**B**) A sequence corresponding to the homologous domain between PKCβ (P05771; residues 218–226) and synaptotagmin-1 (P21579; residues 202–213) was identified, and the βC2-4 peptide corresponding to this sequence was developed. Scheme is not drawn to scale. This figure was created using BioRender.com (accessed on 1 October 2023).

The regulatory domain of the classic PKC isozymes contains two common regions, C1 and C2. While the C1 region is also found in the novel PKC subfamily, the C2 region is present only in the classic PKC subfamily and mediates direct binding to lipids at the plasma membrane [140]. In addition, the C2 region of classic PKC contains at least part of the RACK-binding site on the enzyme. Ron et al. hypothesized that homologous sequences within the C2 region of PKCβ and synaptotagmin-1, a calcium sensor that triggers neurotransmitter release at the synapse, may mediate their binding to RACK1. Initially, they demonstrated that synaptotagmin fragments containing the C2 homologous region bind to purified RACKs and inhibit PKC binding [141]. Next, they designed three peptides derived from the homologous sequences of PKCβ and synaptotagmin, βC2-1 (PKCβ_209-216,_ KQKTKTIK), βC2-2 (PKCβ_186-198,_ MDPNGLSDPYVKL), and βC2-4 (PKCβ_218-226,_ SLNPEWNET, aka αC2-4). In addition, the peptide βC2-3 (PKCβ_201-207,_ IPDPKSE) derived from a region with no homology was also synthesized as control and demonstrated no biological activity. The three homologous peptides bound RACK1 and inhibited the PKC C2 fragment binding. In addition, these peptides specifically inhibited the phorbol-ester-induced translocation of the C2-containing isozymes in cardiac myocytes, as well as the insulin-induced PKCβ translocation and function in *Xenopus oocytes*. Therefore, these peptides act as specific inhibitors for functions mediated by PKCβ (Table 7; Figure 6) [69,142].

Cianciolo et al. compared the sequences of the p15E retroviral transmembrane protein that is conserved across type C and D retroviruses, as well as human T-cell lymphoma viruses (HTLVs). The authors found a high degree of homology (73%) between the p15E, HTLV-I, and HTLV-II proteins, which occurred in the p21 sequence of HTLV where the first ten amino acids are identical [143,144]. Based on this finding, CKS-17 peptide was derived from p15E and conjugated to BSA, carrier protein, (CKS-17-BSA). The conjugate inhibited Ca^2+^- and phosphatidylserine-dependent PKC activity in cell homogenates (Table 7) [145].

**Table 7:** Peptides derived from conserved sequences in homologous domains of otherwise non-related proteins.

Peptide	Parent Protein_AA-AA_	Protein Accession #	Peptide Sequence	Peptide Role
βC2-1	PKCβ_209-216_ homolog to Synaptotagmin-1_193-200_	P05771 P21579	KQKTKTIK : .:: . KFETKVHR	**Inhibitor**: the peptide inhibited classic PKCs binding to RACK [142]. **Antagonist**: the peptide inhibited classic PKC translocation and function [142].
βC2-2	PKCβ_186-198_ homolog to Synaptotagmin-1_174-184_	P05771 P21579	MDPNGLSDPYVKL : : ::::::. M--GGTSDPYVKV	**Inhibitor**: the peptide inhibited classic PKCs binding to RACK [142]. **Antagonist**: the peptide inhibited classic PKC translocation and function [142].
βC2-4	PKCβ_218-226_ homolog to Synaptotagmin-1_202-213_	P05771 P21579	SLNPEWNET .::: :: TLNPVFNEQ	**Inhibitor**: the peptide inhibited classic PKCs binding to RACK [142]. **Antagonist**: the peptide inhibited classic PKC translocation and function [142].
CKS-17	p15E (ENV_539-555_) homolog to ENV HTL1M_376-392_	P03386 P23064	LQNRRGLDLLFLKEGGL .:::::::::: ..::: AQNRRGLDLLFWEQGGL	**Antagonist**: the peptide inhibited PKC activity [145]

Amino acids are represented by their one-letter code; “:” indicates identical amino acids; and “.” indicates conserved amino acid substitutions.

## 6. Peptides Derived from Unique Sequences of the Protein Kinase

Compound specificity is often a major drawback. For example, many kinase SMKIs function as competitive inhibitors of the ATP-binding site that is structurally similar. Therefore, many of them suffer from a lack of selectivity for structurally related kinase families. This results in off-target toxicity that can cause dangerous side effects and is a major cause of clinical trial failure. One approach to overcoming this hurdle is by identifying unique kinase sequences. For example, by aligning the variable domain (V5) of two PKCβ isozymes, Stebbins et al. developed peptides that are not only specific to PKC, but also to one PKC isozyme [146].

### 6.1. Peptides Derived from Unique Sequences in Homologous Domains of Related Proteins

**Figure 7:**
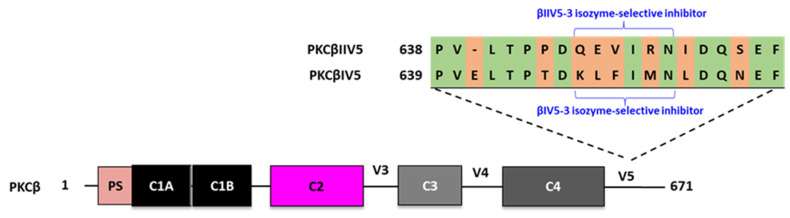
Peptides derived from unique sequences in homologous domains of related proteins. A sequence corresponding to the less homologous domain between PKCβII (P05771-2; residues 645–650) and PKCβI (P05771-1; residues 646–651) was identified, and the βIIV5-3 peptide corresponding to this sequence was developed.

The classic PKCβ isozyme has two alternatively spliced forms (PKCβI and PKCβII), which differ only at their COOH-terminal V5 regions (i.e., last 50 amino acids). Therefore, peptides derived from the V5 region should be isozyme-selective. Stebbins et al. identified that the V5 domain of PKCβII contains part of the RACK1-binding site. Based on that observation, three peptides corresponding to unique regions were selected from each of the βI and βII V5 domains: βIV5-1, βIV5-2, βIV5-3, βIIV5-1, βIIV5-2, and βIIV5-3. The βIIV5-3 peptide selectively inhibited the phorbol 12-myristate 13-acetate (PMA)-induced translocation of PKCβII and not PKCβI in neonatal rat cardiac myocytes and inhibited cardiac myocyte hypertrophy in PMA-treated cells (Figure 7). βIV5-3 also inhibited PMA-induced cardiac myocyte hypertrophy, suggesting that both PKCβ isozymes are essential for this cardiac function [146]. A similar approach was used to rationally design two additional peptides derived from PKCγ and PKCα. The investigators aligned the four classic PKCs (PKCα, PKCβI, PKCβII, and PKCγ) and identified amino acid sequences that vary between the isozymes. Sweitzer et al. developed the γV5-3 peptide derived from PKCγ that selectively inhibits PKCγ. The peptide reduced nociception by blocking the activation of specific neurons in a spinal cord model [147,148,149]. Kim et al. developed the αV5-3 peptide, a novel inhibitor selective for PKCα. In mice, primary tumor growth was not affected by αV5-3 treatment, yet mortality was reduced and metastasis to the lung decreased by more than 90%. In addition, αV5-3 treatment reduced invasion by reducing matrix metalloproteinase-9 activities and decreased tumor cell migration. Finally, the peptide showed superior efficacy relative to antibody treatment in reducing metastasis in vivo (Table 8) [150].

### 6.2. Peptides Derived from Sequences That Are Overlapping with Identified Bioactive Peptides in Homologous Domains of Related Proteins

C2 domains function as calcium-dependent membrane-binding modules in the regulatory domain of many proteins that participate in membrane trafficking and signal transduction. These domains share a common tertiary structure comprising eight anti-parallel β-strands connected by variable loops. Importantly, their inter- and intra-molecular interactions are vital for PKC activation, translocation, and PPIs (e.g., binding to substrates and anchoring proteins). Several peptides were designed based on the similarity to a bioactive peptide derived from the C2 domain. Using molecular modeling and structural homology analyses, Chen et al. developed the PKCδ-selective inhibitor δV1-1 peptide. δV1-1 is derived from a structural similarity between the secondary structure of δV1 [153] and the domain of εV1-2 located on the modeled secondary structure of εV1. δV1-1 protected isolated hearts from ischemic damage [119,154]. Using a similar approach based on sequence similarity, ηV1-2, a novel peptide, was also developed and was demonstrated to be a PKCη antagonist (Table 9) [134,155].

### 6.3. Peptides Derived from Additional Critical Kinase Domains

A peptide corresponding to the caspase-3 cleavage site of PKCδ was developed as an antagonist. The authors hypothesized that this peptide would prevent the caspase-3-mediated cleavage and activation of PKCδ. The z-DIPD-fmk peptide was more potent than the most widely used and commercially available caspase-3 inhibitor (IC_50_ 6 μM) and effectively blocked PKCδ cleavage and proteolytic activation (Table 10) [157].

A peptide inhibitor of the nuclear translocation site was also developed for another PKC isozyme. The authors hypothesized that molecules mimicking the canonical PKCθ nuclear localization signals (NLSs) would act as a specific competitive inhibitor. Indeed, the peptide inhibited PKCθ translocation without affecting its catalytic activity (Table 10) [158,159].

## 7. Summary

Inhibitors of protein kinases are becoming increasingly represented in the therapeutic arsenal for a variety of indications, especially in oncology. Although targeted PKC modulators are only a few of the approved kinase inhibitors to date, many have been trialed clinically and even more are in clinical development. It is clear that PKC functions as a master regulator across numerous pathological processes, in which different PKC isozymes play distinct roles. The breadth of biological processes across which PKC isozymes mediate signaling presents major challenges for PKC inhibitors due to severe toxicities seen for molecules without sufficient isozyme selectivity and specificity.

Recent efforts have focused on allosteric modulators to achieve better PKC isozyme selectivity and drug-like properties. Traditional small molecules have been less useful for this application due to the large interacting services without defined binding pockets that characterize the PPIs that take place at allosteric binding sites [160]. Antibody therapeutics are not particularly convenient for targeting PKC given the intracellular target location, as well as production and dosing difficulties. Peptides are a promising alternative approach being leveraged as allosteric modulators to selectively target PKC isozymes (thereby reducing potential toxicity) due to their unique capacity to bind large PPI interacting surfaces, permeate the cell membrane, and ease production concerns. Further, advanced techniques are now readily available for modification of peptide leads to create peptidomimetics with enhanced metabolic and conformational stability, as well as other desirable drug properties. Based on these advances and an improved understanding of PKC signaling, we foresee more peptide-based compounds entering development and clinical studies for the modulation of PKC targets.

## Figures and Tables

**Table 5:** Peptides derived from sequences involved in intramolecular interactions.

Peptide	Parent Protein_AA-AA_	Protein Accession #	Peptide Sequence	Peptide Role
ψRACK1 (ψβRACK)	PKCβ_241-246_ homolog to RACK1_255-260_	P05771 P63244	SV**E**IWD :..::: SI**K**IWD	**Inhibitor**: the peptide inhibited PKCβ binding to RACK [131]. **Agonist**: the peptide activates PKCβ in the absence of PKC activators [131].
ψεRACK (εV1-7)	PKCε_85–92_ homolog to β′-COP_285-292_	Q02156 P35606	HDAPIGYD . .::: NNVALGYD	**Agonist**: the peptide increased PKCε translocation and function [132].
ψδRACK	PKCδ_71-83 (*74-81 underlined*)_ homolog to Annexin V_154-166_	Q05655 P08758	IVLMRRA**E**DPMSE .:: .: .:: VVLLQAN**R**DPDAG	**Agonist**: the peptide increased PKCδ translocation and function [119].

Amino acids are represented by their one-letter code; “:” indicates identical amino acids; and “.” Indicates conserved amino acid substitutions.

**Table 6:** Peptides derived from evolutionarily conserved sequences.

Peptide	Parent Protein_AA-AA_	Protein Accession #	Peptide Sequence	Peptide Role
εV1-2	PKCε_14-21_ (human) homolog to PKCε_19-26_ (slug)	Q02156 Q16975	EAVSLKPT ::: :::: EAVDLKPT	**Inhibitor**: the peptide inhibited PKCε binding to RACK [139]. **Antagonist**: the peptide inhibited PKCε translocation and function [134].
ψεRACK (εV1-7)	PKCε_85-92_ homolog to PKCε_84-91_ (Slug)	Q02156 Q16975	HDAPIGYD ::: : : HDAAIPPD	**Agonist**: the peptide increased PKCε translocation and function [132].

The peptides above were developed based on sequence homology between isozymes, in a manner analogous to that depicted in Figure 4. Amino acids are represented by their one-letter code; “:” indicates identical amino acids.

**Table 8:** Peptides derived from unique sequences in homologous domains of related proteins.

Peptide	Parent Protein_AA-AA_	Protein Accession #	Peptide Sequence	Peptide Role
βIV5-3	PKCβI_646-651_ *least similar to* PKCβII_645-650_	P05771-1 P05771-2	KLFIMN . : QEVIRN	**Antagonist**: the peptide inhibited PKCβI translocation and function [146].
βIIV5-1	PKCβII_660-673_ *least similar to* PKCβI_661-671_	P05771-2 P05771-1	SFVNSEFLKPEVKS :. : ::. SYTNPEFVINV---	**Inhibitor**: the peptide partially inhibited PKCβII binding to RACK [146]. **Antagonist**: the peptide inhibited PKCβII function [151,152].
βIIV5-2	PKCβII_621-627_ *least similar to* PKCβI_621-627_	P05771-2 P05771-1	ACGRNAE : . ARDKRDT	**Inhibitor**: the peptide partially inhibited PKCβII binding to RACK [146].
βIIV5-3	PKCβII_645-650_ *least similar to* PKCβI_646-651_	P05771-2 P05771-1	QEVIRN . : KLFIMN	**Inhibitor**: the peptide partially inhibited PKCβII binding to RACK [146]. **Antagonist**: the peptide inhibited PKCβII translocation and function [146].
γV5-3	PKCγ_659-664_ *least similar to* PKCβII_645-650_	P05129 P05771-2	RLVLAS . :. QEVIRN	**Antagonist**: the peptide inhibited PKCγ translocation and function [147,148,149].
αV5-3	PKCα_642-647_ *least similar to* PKCβII_645-650_	P17252 P05771-2	QLVIAN : :: : QEVIRN	**Antagonist**: the peptide inhibited PKCγ translocation and function [150].

Amino acids are represented by their one-letter code; “:” indicates identical amino acids; and “.” indicates conserved amino acid substitutions.

**Table 9:** Peptides derived from sequences that are overlapping with identified bioactive peptides in homologous domains of related proteins.

Peptide	Parent Protein_AA-AA_	Protein Accession #	Peptide Sequence	Peptide Role
δV1-1 (KAI-9803)	PKCδ_8–17_	Q05655	SFNSYELGSL	**Antagonist**: the peptide inhibited PKCδ translocation and function [119].
ηV1-2	PKCη_18-25_	P24723	EAVGLQPT	**Antagonist**: the peptide inhibited PKCη translocation and function [156].

The peptides above were developed based on sequence homology between isozymes, in a manner analogous to that depicted in Figure 4.

**Table 10:** Peptides derived from additional critical kinase domains.

Peptide	Parent Protein_AA-AA_	Protein Accession #	Peptide Sequence	Peptide Role
z-DIPD-fmk	PKCδ_324-327_	Q05655	DIPD	**Antagonist**: the peptide inhibited PKCδ function [157].
PKCθ	PKCθ_644-656_	Q04759	RKEIDPPFRPKVK	**Antagonist**: the peptide inhibited PKCθ translocation [158].
